# Socioeconomic analysis of the in-hospital neurosurgical continuum of care for patients in Nigeria

**DOI:** 10.3389/fneur.2026.1897423

**Published:** 2026-10-23

**Authors:** Amos O. Adeleye, Allan Bakesiga, Oluseyi O. Agboola, Claudia Fernandez Perez, Prince Antwi, Benjamin Mukumbya, Chibueze Agwu, Ifeanyichukwu Ozobu, Mohamed-Yahia Monawar, C. Maxwell Beck, Madison Evans, Kabiru K. Salami, Adebola E. Orimadegun, Osondu Ogbuoji, Michael M. Haglund, Alvan-Emeka K. Ukachukwu

**Affiliations:** 1 Department of Surgery, College of Medicine, University of Ibadan, Ibadan, Nigeria; 2 Department of Neurological Surgery, University College Hospital, Ibadan, Nigeria; 3 Duke Global Neurosurgery and Neurology, Durham, NC, United States; 4 Duke Global Health Institute, Durham, NC, United States; 5 Georgetown University School of Medicine, Washington, DC, United States; 6 Department of Neurosurgery, Duke University Health System, Durham, NC, United States; 7 University of Chicago Pritzker School of Medicine, Chicago, IL, United States; 8 Duke University School of Medicine, Durham, NC, United States; 9 University of Maryland School of Medicine, Baltimore, MD, United States; 10 Department of Sociology (Medical Sociology), University of Ibadan, Ibadan, Nigeria; 11 Institute of Child Health, College of Medicine, University of Ibadan and University College Hospital, Ibadan, Nigeria

**Keywords:** catastrophic health expenditure, financial risk protection, health inequities, healthcare financing, neurosurgery, Nigeria, out-of-pocket (OOP) expenditure

## Abstract

**Background:**

In Nigeria, neurosurgical care is primarily financed by out-of-pocket (OOP) payments due to minimal health insurance coverage, leading to high rates of financial distress, limited access, and delayed care. This study aimed to quantify patient-level OOP costs, evaluate the risk of catastrophic and impoverishing expenditures, and assess the socioeconomic impacts of neurosurgical hospitalization.

**Methods:**

We conducted a prospective cohort study of neurosurgical patients at the University College Hospital, Ibadan, from January to August 2024. Structured questionnaires were administered at admission, discharge, and 2–4 weeks post-discharge to capture direct medical, direct non-medical, and indirect costs of care. Catastrophic health expenditure (CHE) was assessed using two established thresholds: out-of-pocket health expenditure exceeding 10% of total household income, consistent with the SDG 3.8.2 framework, and expenditure ≥40% of household capacity to pay, using the WHO approach. Impoverishment was assessed against the World Bank poverty line for Nigeria. Ethical approvals were obtained from the University of Ibadan/University College Hospital Ibadan and Duke Health Institutional Review Boards.

**Results:**

We enrolled 99 neurosurgical patients (median age 33, 75% male). Traumatic brain injury was the most common diagnosis (48%). The median length of stay was 14 days. Most patients (92%) relied on household savings, and 52% borrowed money to cover their cost of care. Only 12% had any health insurance, which covered a median of 30% of expenses. The median direct medical cost per patient was ₦969,000 ($633), non-medical cost ₦202,000 ($132), and indirect cost ₦141,639 ($93). The median total OOP expenditure was ₦1,410,787 ($922; IQR: ₦815,951–₦2,442,176). Among households with sufficient baseline economic data, 97.0% were already below the specified poverty threshold before healthcare payments. Both households initially above the threshold crossed below it, following OOP expenditure, while the mean daily per-capita poverty gap increased from ₦3,296 to ₦30,350.

**Conclusion:**

These findings underscore the inadequacy of current financial protection mechanisms. To improve access and equity, urgent policy action is needed to expand pooled health financing, increase public investment in neurosurgical services, and provide targeted subsidies for high-cost surgical care. Strengthening public neurosurgical services and decentralizing care (to reduce travel and indirect costs) will also help shield vulnerable households. These measures are critical as Nigeria advances toward universal health coverage.

## Introduction

Neurosurgical care is inherently resource-intensive, requiring substantial financial, infrastructural, and human capital. In many low- and middle-income countries (LMICs), such as Nigeria, where over 70% of the population resides in rural areas, access to such care remains limited and often unaffordable. Despite being Africa’s most populous nation, Nigeria continues to grapple with poor health indices, an underfunded healthcare system, and very limited insurance coverage. Out-of-pocket (OOP) expenditure constitutes the dominant means of financing healthcare, accounting for approximately 74% of health spending, with nearly half of households experiencing catastrophic health expenditure (CHE) (1, 2). This is particularly prominent in more specialized fields like neurosurgery, where the cost of diagnostics, surgical intervention, intensive care, and rehabilitation is, as a rule, substantially higher than what most other in-hospital healthcare costs are, even in more advanced health systems. Consequently, it is unsurprising that these costs far exceed what already impoverished households in LMICs can afford to pay OOP (3).

While the public sector is subsidized to some extent, for instance, in terms of workforce salaries, the in-hospital journey of most patients still relies heavily on patient-sourced payments for health service delivery, including service charges and procurement of hospital consumables. Moreover, although Nigeria has a National Health Insurance Scheme (NHIS), this system is yet not well-developed, and covers fewer than 5% of the population, primarily benefiting virtually only those employed in the formal public service sector, who represent less than 10% of the national population (4–6). As a result, many patients presenting with any complex medical and or neurosurgical conditions – including traumatic brain injury (TBI), congenital anomalies, degenerative spine disorders, tumors, and cerebrovascular diseases – are forced to fund their care privately. This often results in delayed presentations, incomplete treatments, or reliance on charitable support and institutional waivers (7). This has significant social and economic consequences for patients and their families, as well as detrimental effects on their overall health (7, 8).

Although the economic burden of healthcare and neurosurgery, specifically in Nigeria, has been described in some contexts, there is a noticeable scarcity of data on the specific costs and socioeconomic consequences of neurosurgical care for patients. Previous studies have evaluated single procedures or narrow cost components within hospital settings, but not the comprehensive patient-borne cost of neurosurgical hospitalization across the entire care pathway (9, 10). Without comprehensive data on patient-level financial burdens, including transportation, lost income, caregiver responsibilities, and psychosocial disruption, it remains difficult to advocate for meaningful health policy reform or resource allocation in neurosurgical services.

To address this critical gap, we conducted a prospective cohort study at the University College Hospital (UCH), Ibadan, Nigeria’s premier tertiary medical center and the oldest neurosurgical unit in West Africa. Our objectives were (1) to quantify the direct and indirect OOP expenditures borne by neurosurgical patients, (2) to determine the proportion of patients experiencing catastrophic and impoverishing healthcare costs, and (3) to characterize the socioeconomic impact of neurosurgical hospitalization on patients and their families. By capturing patient-reported data from admission through 30 days post-discharge, this study provides a holistic view of the financial burdens of neurosurgical care in a resource-limited setting. The findings aim to inform health financing reforms and drive more equitable access to essential surgical care.

## Methods

### Study design and setting

We conducted a prospective mixed-methods study, using a quantitative survey and qualitative interviews at UCH Ibadan, a tertiary referral and teaching hospital in Southwest Nigeria. While the results of the quantitative survey are reported here, details of the qualitative arm are described in an accompanying manuscript.

### Study population

Eligible participants included all adult and pediatric patients admitted to the neurosurgery department between January and August 2024. Caregivers of patients who were less than 18 years of age, comatose, with marked intellectual disability, or with physical challenges that precluded them from completing the survey served as proxy respondents. Patients were followed from admission, through discharge, and up to 30 days post-discharge to capture ongoing costs.

### Data collection

Trained research assistants administered structured questionnaires hosted on Qualtrics *(Qualtrics XM, Provo, UT, USA)* at three time points: within 24 h of admission, within 48 h of discharge, and 2–4 weeks post-discharge via in-person home visits or phone calls. The survey collected data on patient demographics, clinical diagnosis, and detailed cost information, including direct medical, direct non-medical, and indirect costs of care. The impact of the cost of care on patients, caregivers, and/or their families was also assessed. We recorded whether participants had any health insurance and the percentage of costs it covered.

### Data analysis

Data were analyzed using R version 4.4.2 *(R Foundation for Statistical Computing, Vienna, Austria)*. Categorical variables were summarized using frequencies and percentages, and continuous variables by median and interquartile ranges (IQR). Direct medical costs included imaging, laboratory tests, medications, consumables, procedures, rehabilitation, surgery, anesthesia, ICU, and medical devices. Direct non-medical costs encompass patient and caregiver transportation, food and lodging during hospitalization and transit, and miscellaneous supplies (toiletries, etc.). Indirect costs were estimated from lost income: we recorded the number of workdays missed by patients and caregivers due to hospitalization and multiplied by reported daily earnings. All expenditures were calculated in Nigerian Naira (₦) and converted to US dollars (USD) for reporting (exchange rate ~₦1,531/USD at the time of study).


$$\begin{array}{l} \text{Total cost of care}=\text{Direct medical cost}+\text{Direct non}\\ -\text{medical cost}+\text{Indirect cost} \end{array}$$

$$\begin{array}{l} \text{Total}\;\mathrm{OOP}=\text{Total cost of care}\;x\\ \;\;\;\;\;\;\;\;\;\;\;\;\;(1-\text{Proportion covered}\;\mathrm{by}\;\text{insurance}) \end{array}$$

We assessed CHE using two thresholds: OOP health expenditure >10% of total household income (CHE10%), consistent with the SDG 3.8.2 framework, and OOP health expenditure ≥40% of non-food household expenditure (CHE40%), based on the WHO capacity-to-pay framework (11, 12).


$$\mathrm{CHE}10\%=\frac{\text{Total out}-\text{of}-\text{pocket expenditure}}{\text{Total monthly household expenditure}}\times 100\%$$

$$\mathrm{CHE}40\%=\frac{\text{Total Out}-\text{of}-\text{Pocket Expenditure}}{\begin{array}{l} \text{Total monthly household expenditure}\\ -\text{Monthly food expenditure} \end{array}}\times 100\%$$

A household was classified as facing CHE if either threshold was exceeded.

Impoverishment was defined as a reduction in daily per capita income below the World Bank’s international poverty line of $3 per person per day for LMICs (equivalent to ₦4,593/day), because of OOP health payments (13, 14). A household was considered impoverished if:


$$\frac{\text{Monthly}\;\text{income}-\mathrm{OOP}\;e\text{xpenditure}}{30\times \text{Household}\;s\mathrm{ize}}<\text{\$}3.$$

Baseline poverty status was determined by comparing pre-payment daily per-capita household income with the specified poverty threshold. Incident impoverishment was defined as crossing from above the poverty threshold before OOP healthcare payments to below the threshold following payment. Among households already below the threshold, poverty deepening was assessed using the poverty gap, calculated as the difference between the poverty threshold and daily per-capita household income before and after OOP expenditure.

## Results

### Participant characteristics

A total of 99 respondents participated in the survey, the majority being caregivers (82.8%, *n* = 82). Among the principal caregivers were parents (39%, *n* = 32), spouses (24.4%, *n* = 20), and adult children (17.1%, *n* = 14). Most patients enrolled were male (74.7%, *n* = 74), with a median age of 33 years (IQR: 14–47). Education levels varied, with the majority (39.4%, *n* = 39) having completed secondary school. The median household size was 5 (IQR: 3–6) (Table 1).

**Table 1:** Sociodemographic characteristics of participants.

Respondent	*N* (%)
Patient	17 (17.2)
Caregiver	82 (82.8)
Gender	
Male	74 (74.7)
Female	25 (25.3)
Caregiver type	
Parent	32 (39.0)
Spouse	20 (24.4)
Child	14 (17.1)
Sibling	11 (13.4)
Other^1^	5 (6.0)
Highest education	
No education	8 (8.1)
Kindergarten	1 (1)
Primary	17 (17.2)
Secondary	39 (39.4)
Tertiary	30 (30.3)
Post-graduate	4 (4)
Occupation	
Student/Dependent	35 (35.4)
Civil servant	10 (10.1)
Self-employed	10 (10.1)
Trader	8 (8.1)
Manual labor	8 (8.1)
Teacher	6 (6.1)
Retired	5 (5.1)
Driver	4 (4.1)
Other^2^	9 (9)
Missing	4 (4.1)

Other^1^, friend, grandparent, cousin, aunt; Other^2^, religious, commercial motorcyclist, healthcare, manager.

Geographically, the majority resided in Oyo State (*n* = 73, 73.7%), where UCH is located, followed by other southwestern states (Ogun: *n* = 12, 12.1%; and Ondo: *n* = 5, 5.1%) (Figure 1). Travel distances varied: 15.2% (*n* = 15) lived within 5 km of the hospital, 41.4% (*n* = 41) between 5–20 km, and 26.3% (*n* = 26) traveled more than 50 km. Despite referrals for most respondents (78.8%, *n* = 78), 66.7% (*n* = 66) reported transit times under one hour (Figure 2).

**Figure 1:**
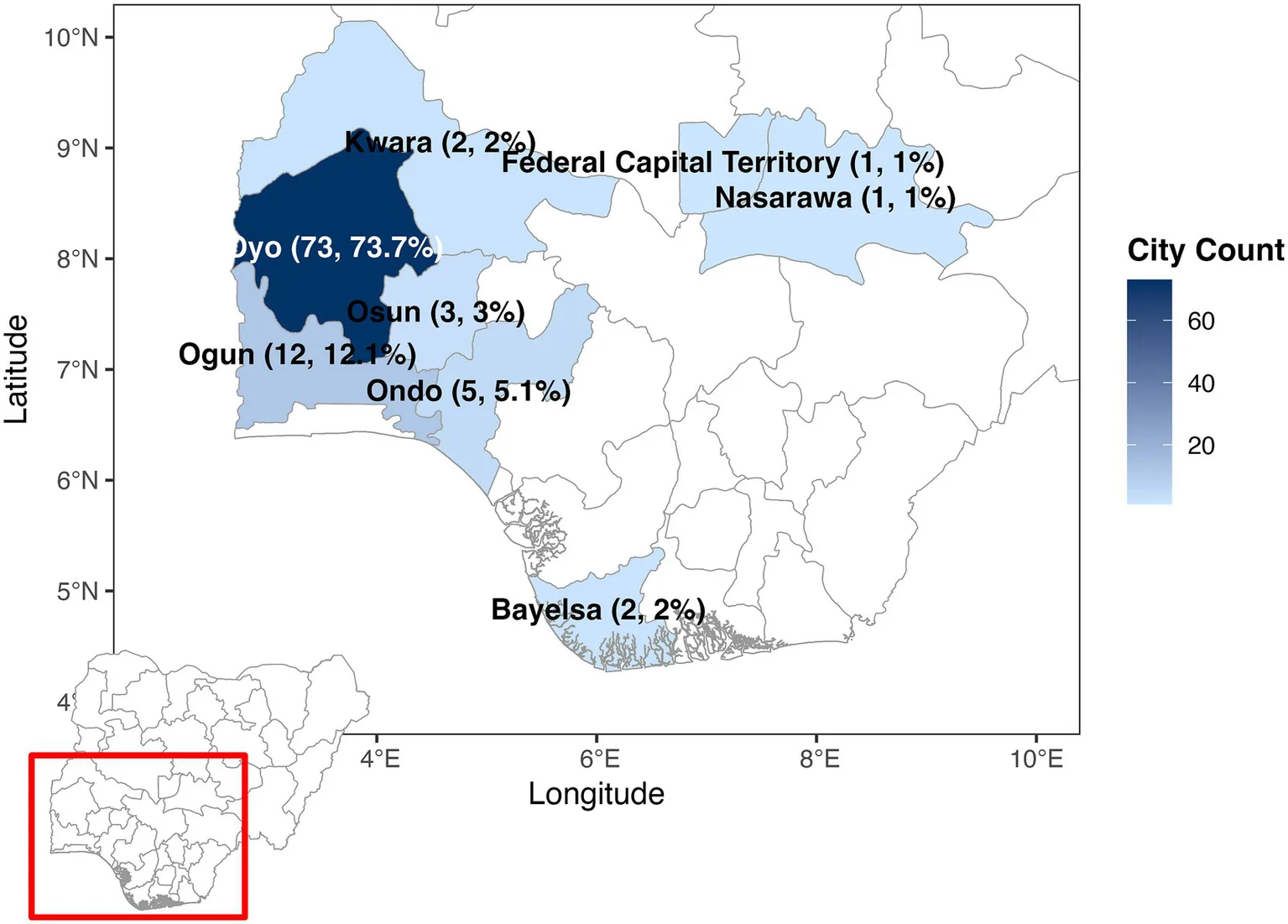
A map illustrating the distribution of participants’ home addresses across Nigerian states, shaded by count and percentage. The inset map provides national context, highlighting the zoomed-in southwestern region where majority of the participants resided.

**Figure 2:**
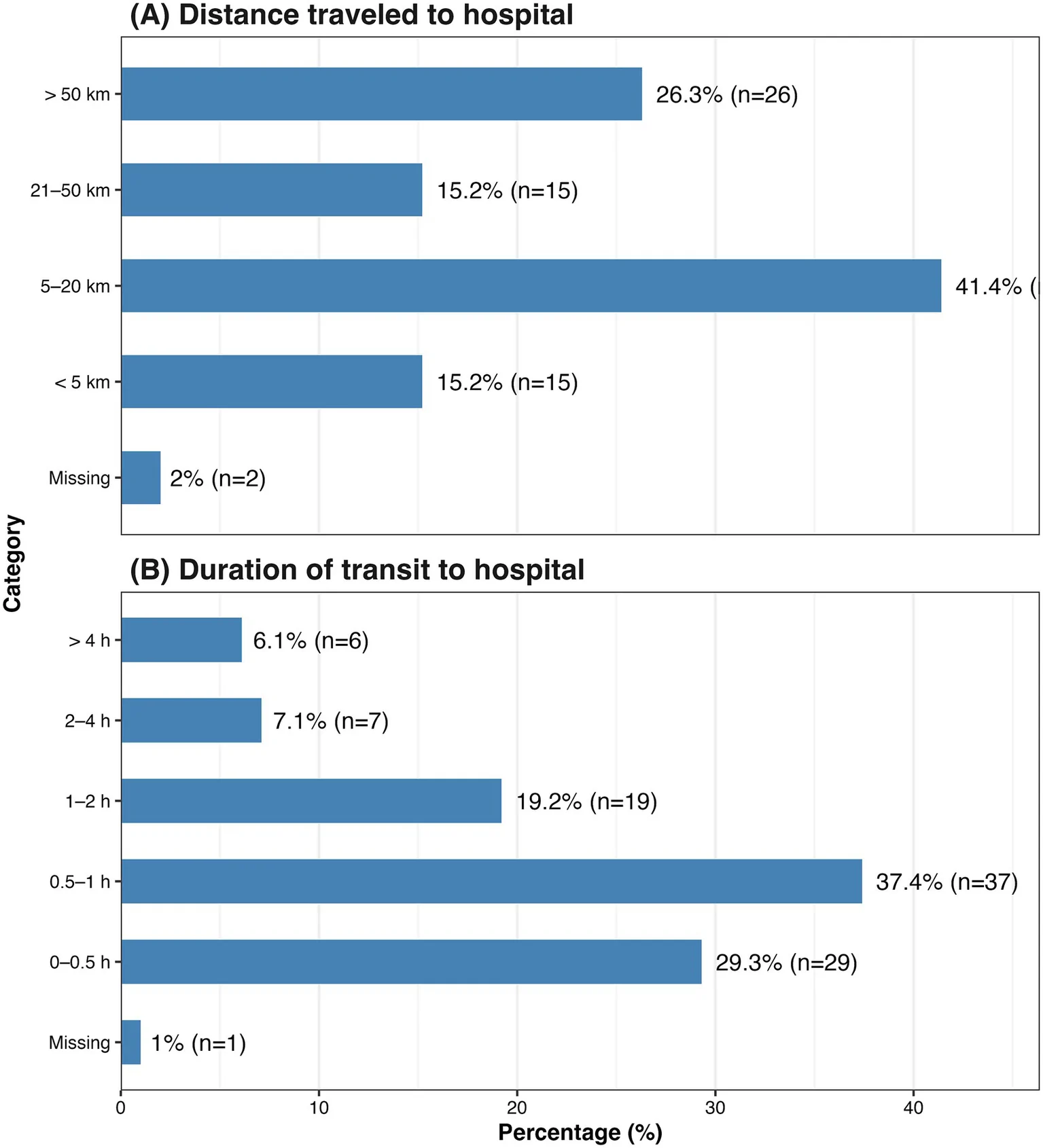
Distribution of distance traveled to the hospital **(A)** and duration of transit to the hospital **(B)**, shown as percentages with corresponding participant counts.

The most common neurosurgical diagnoses were traumatic brain injury (TBI, 48%, *n* = 47), brain tumors (20%, *n* = 20), and hydrocephalus (8.2%, *n* = 8). The overall median hospital stay was 14 days (IQR: 8–24.75), with TBI patients staying a median of 10 days (IQR: 7–15), brain tumors 20 days (IQR: 11.25–31.25), and hydrocephalus 13.5 days (2.75–18.25) (Figure 3). Longer stays were often linked to intensive care needs or rehabilitation.

**Figure 3:**
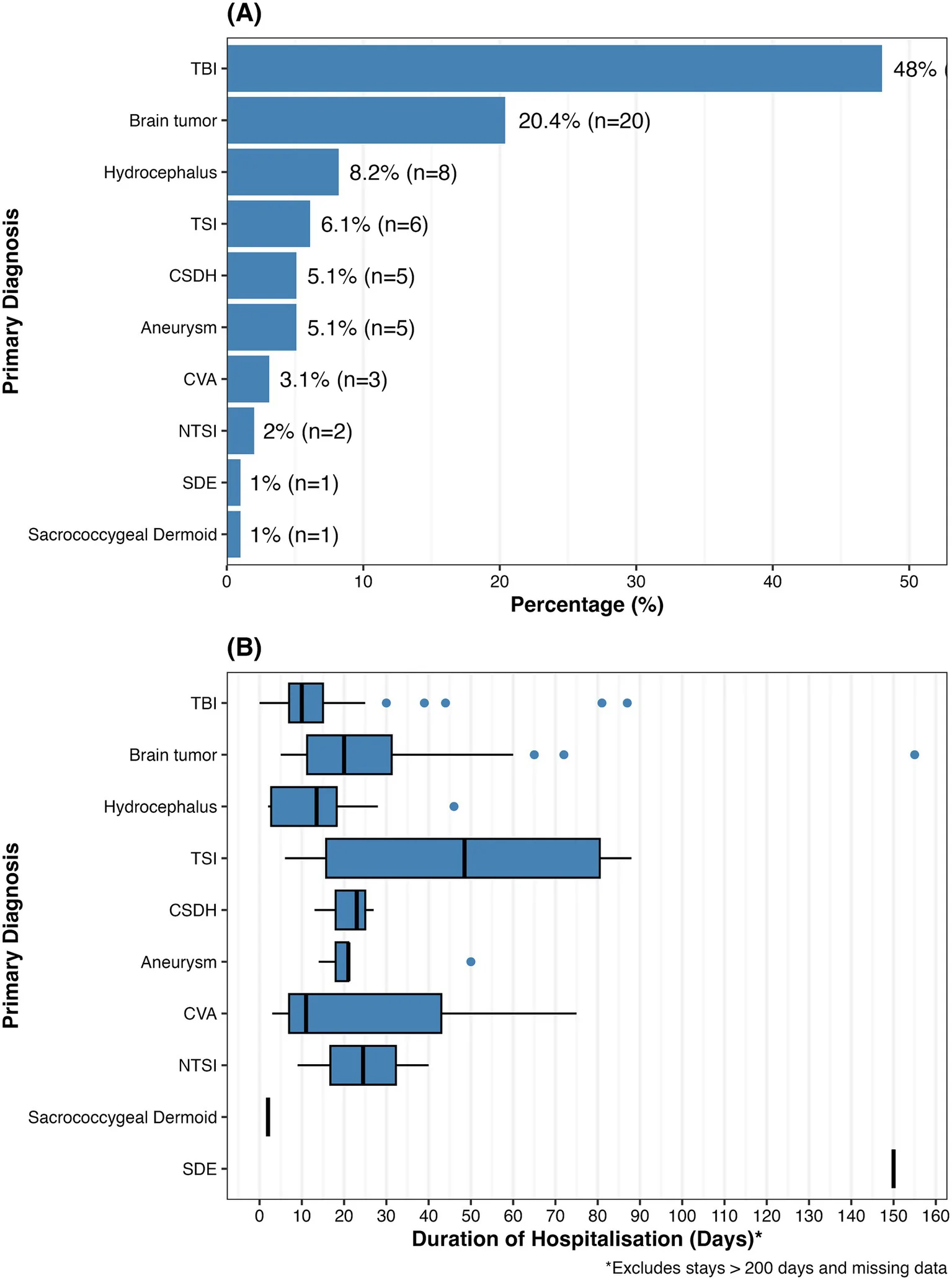
Distribution of primary diagnoses **(A)** and hospital stay duration by diagnosis **(B)**. Hospitalization duration >200 days–one patient with TSI – was excluded. CSDH, Chronic subdural hematoma; CVA, Cerebral vascular accident; NTSI, Non-traumatic spine injury; SDE, Subdural empyema; TBI, Traumatic brain injury; TSI, Traumatic spine injury.

### Household expenditure

Most households (56.4%, *n* = 53) reported monthly expenses between ₦100,000 and ₦250,000 (~ $65–$163) (Figure 4). Only 12.1% (*n* = 12) of patients had any health insurance (all via NHIS), and among these, the median proportion of costs covered was 30% (IQR: 16–70%).

**Figure 4:**
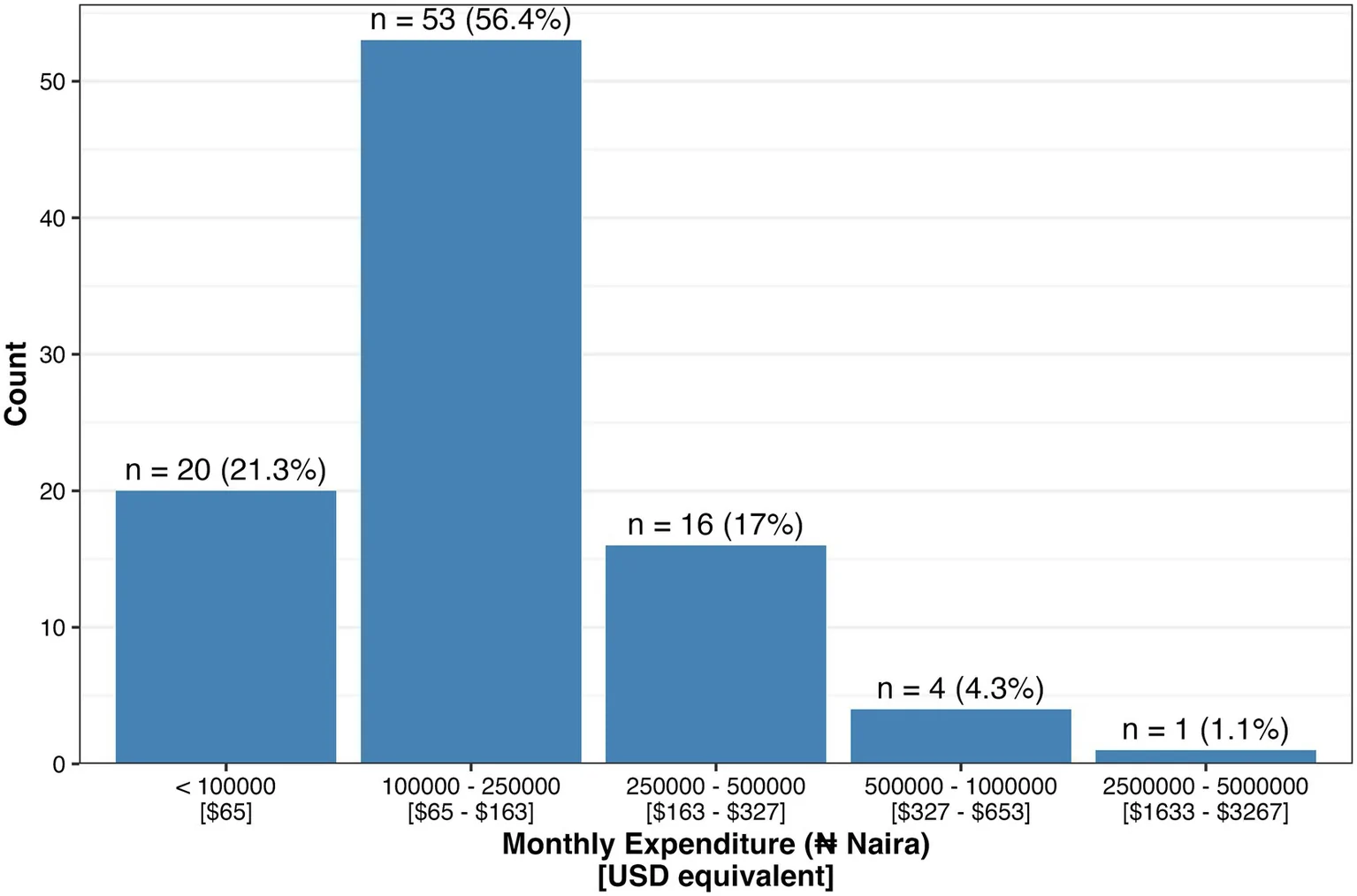
Distribution of patients’ monthly living expenses.

Figure 5 displays the flow of a household’s median monthly income (₦100,000 [$65]) through major expenditure categories, including food, rent, childcare, leisure, and healthcare, and shows the resulting income deficit (₦55,000 [$36]). The figure also depicts the median OOP cost for a neurosurgical admission (₦1,410,787 [$922]), separating the portion attributable to routine healthcare spending (₦10,000 [$7]) from the remaining OOP cost deficit (₦1,400,787 [~$915]).

**Figure 5:**
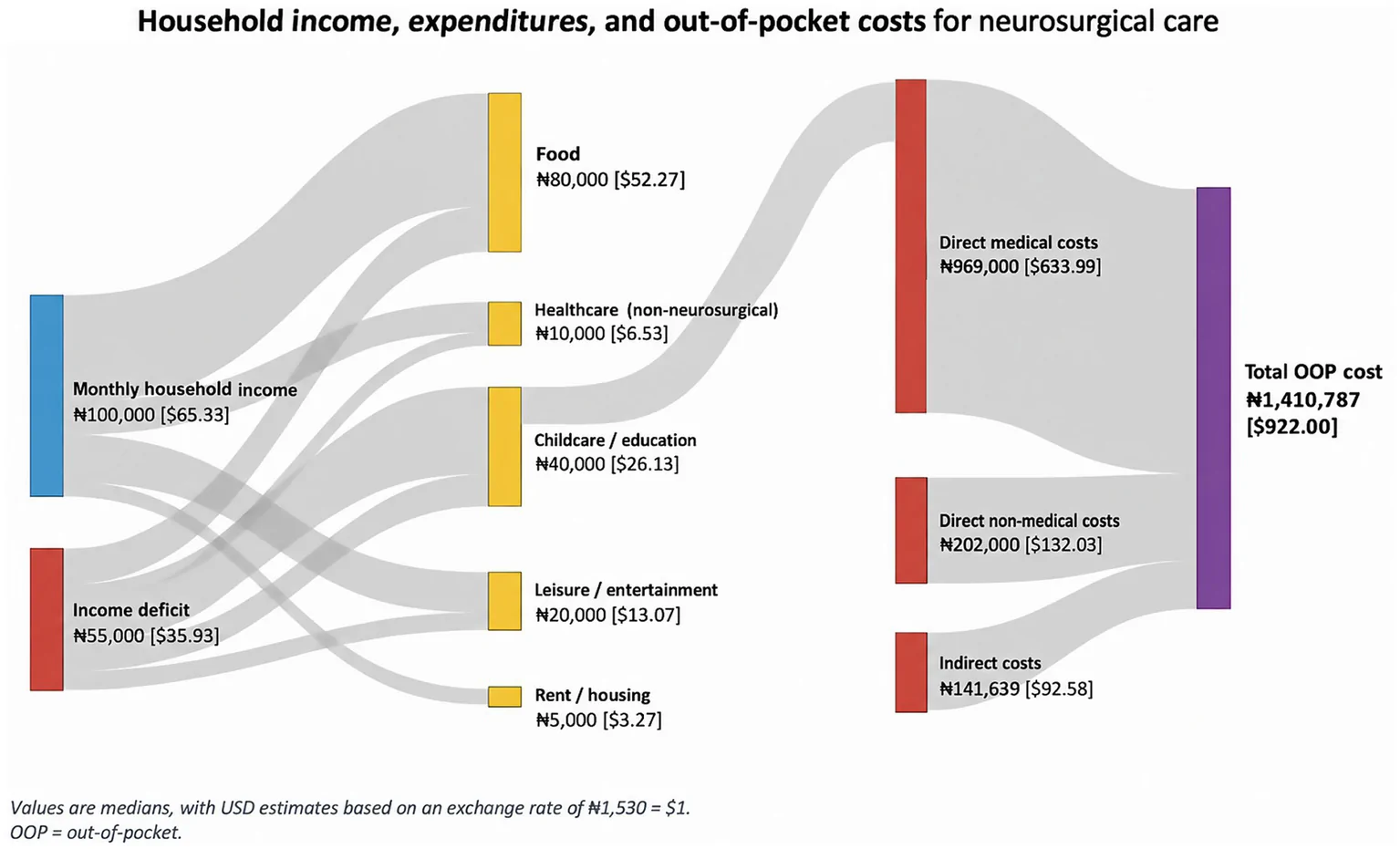
Sankey diagram showing the flow of median household income, median routine expenditures, and median out-of-pocket costs for neurosurgical care.

### Out-of-pocket expenditures

Direct medical costs were the largest component of spending. The median total direct medical expense per patient was ₦969,000 ($633; IQR: ₦507,342–₦1,457,635). Major contributors included surgery/procedure fees (median ₦280,000 [$183] for those undergoing surgery), medications (₦150,000 [$98]), and consumables (₦60,000 [$39]). In addition, non-medical costs were substantial: the median total transportation, food, and lodging costs while hospitalized was ₦202,000 ($132; IQR: ₦97,750–₦308,700). Indirect costs from lost income had a median of ₦141,639 ($93; IQR: ₦41,902–₦539,180). After accounting for direct medical, direct non-medical, and indirect costs, the median total OOP expenditure per patient was ₦1,410,787 ($922; IQR: ₦815,951–₦2,442,176) (Table 2; Figure 6; Appendix). Patients who required intensive care or advanced devices tended to report the highest bills.

**Table 2:** Distribution of out-of-pocket expenditures by cost category.

Category	*n* (%)	Median (₦) (IQR)	Median ($) (IQR)
Direct medical costs	99 (100%)	₦969,000 (₦507,342–₦1,457,635)	$633 ($331–$952)
Direct non-medical costs	95 (96.0%)	₦202,000 (₦97,750–₦308,700)	$132 ($64–$202)
Indirect costs	88 (88.9%)	₦141,639 (₦41,902–₦539,180)	$93 ($27–$352)
Total OOP cost	99 (100%)	₦1,410,787 (₦815,951–₦2,442,176)	$922 ($533–$1,596)

**Figure 6:**
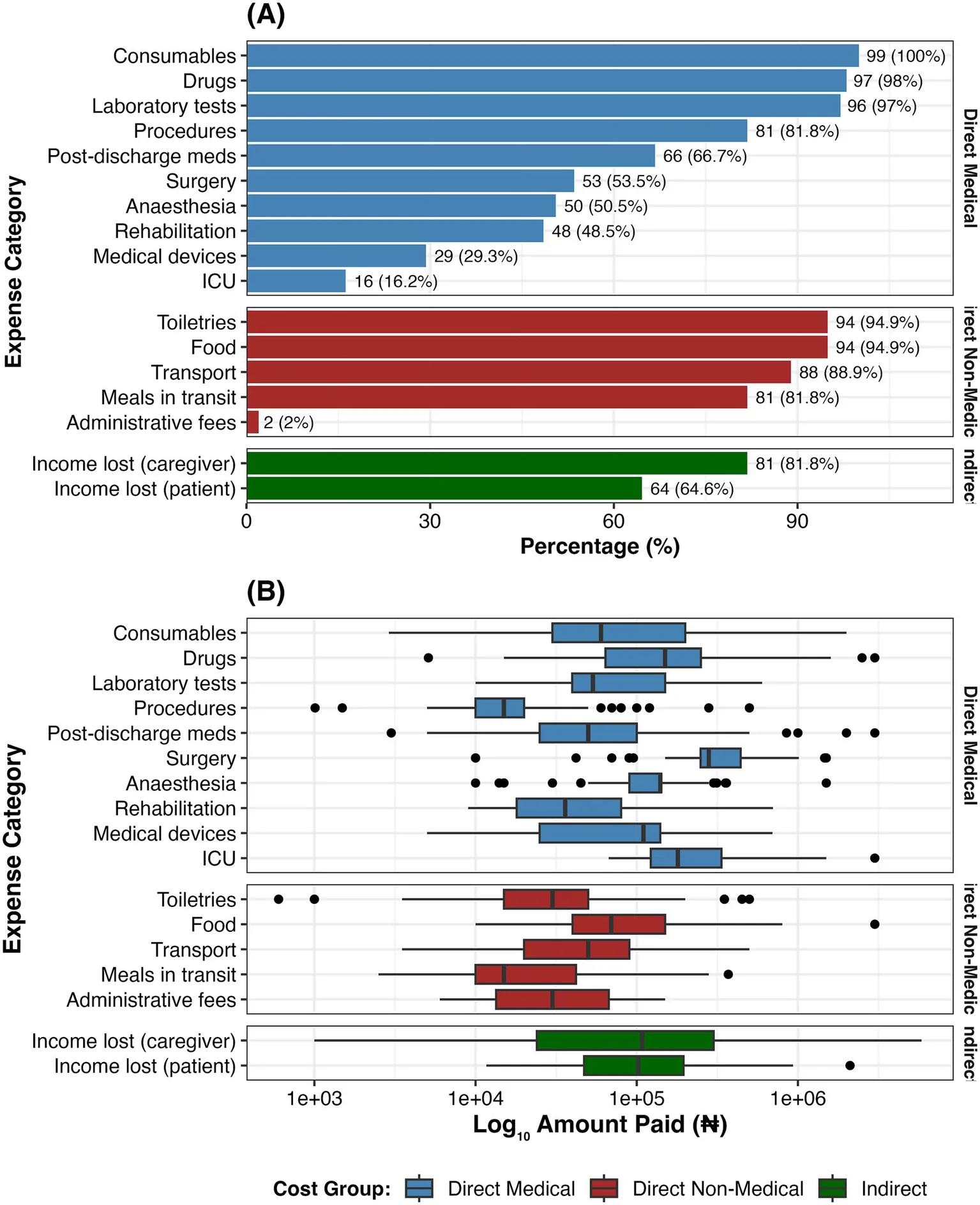
Bar chart **(A)** shows the proportion of respondents with OOP payments; boxplot **(B)** displays the distribution of OOP amounts on a log_10_ scale, both grouped by cost category.

Notably, this outlay often exceeded several months’ household income. 92% of participants reported using personal or household savings to cover OOP expenses. Over half of families (51.5%, *n* = 51) borrowed money to cover medical costs, with a median borrowed amount of ₦700,000 ($457; IQR: ₦350,000–₦1,350,000). Among these, 74.5% (*n* = 38) incurred interest on the loan, with a median interest amount of ₦100,000 ($65; IQR: ₦47,500–₦177,500). Additionally, 9.1% (*n* = 9) sold assets (such as land or equipment) to fund their medical expenses. Similarly, 86.9% (*n* = 86) received financial support from others, mostly from family members (89.5%, *n* = 77), followed by religious organizations (27.9%, *n* = 24) and friends (24.4%, *n* = 21). Only 4% received charitable assistance. It took a median of 5 days (IQR: 1–14) for the households to raise the necessary funds to cover the medical expenses. Many patients (89.9%, *n* = 89) received help from a relative with childcare or household chores during their hospital stay. Patients missed a median of 19 household or childcare days, while caregivers missed a median of 20 workdays. The majority (86%, *n* = 61) were not compensated for these missed days, while 4.1% (*n* = 3) received partial compensation and 9.9% (*n* = 7) were fully compensated.

### Catastrophic expenditure and impoverishment

Among 67 households with sufficient income and household-size data, 65 (97.0%) were already below the specified poverty threshold before OOP healthcare payments. Both households above the threshold at baseline crossed below it, following OOP expenditure, corresponding to incident impoverishment in 2/67 (3.0%) of the analyzable cohort. The mean daily per-capita poverty gap increased from ₦3,296 before OOP expenditure to ₦30,350 following OOP expenditure, indicating substantial deepening of pre-existing poverty.

## Discussion

Importantly, the financial impact observed in this cohort was predominantly characterized by the deepening of pre-existing poverty rather than incident impoverishment. Among households with sufficient baseline economic data, 97.0% were already below the specified poverty threshold before OOP healthcare payments. Although both households initially above the threshold subsequently crossed below it, the more striking finding was the substantial increase in the mean daily per-capita poverty gap, from ₦3,296 before OOP expenditure to ₦30,350 following payment. These findings indicate that neurosurgical costs are being borne by households with profound baseline economic vulnerability and further deepen existing deprivation.”

The median OOP expenditure per patient exceeded ₦1.4 million ($922), in a context where more than half of the households reported monthly total household expenditures below ₦250,000 ($163). Health insurance coverage was limited and inadequate, with only 12.1% of patients reported having any insurance, and among those, the median proportion of costs covered was 30%. These findings are consistent with prior work from Nigeria, which revealed that over 90% of individuals pay for healthcare OOP and that awareness and enrollment in the NHIS remain critically low, <5% (15–17). Compared to similar studies, which reported CHE rates ranging from 21.7 to 46.8% and impoverishment rates between 24.3 and 30.9% among patients with non-communicable diseases in public and private health facilities, the financial toll of neurosurgical care in our study is disproportionately higher (18, 19). According to IHME projections, OOP spending will still account for 71.2% of Nigeria’s total health expenditure in 2025, reflecting a system where households remain the primary financiers of care (20). This national-level pattern is mirrored in our study, which illustrates how routine household income is quickly exhausted by basic expenditures, leaving families unable to absorb the cost of a single neurosurgical admission and forcing them to cover nearly the entire OOP burden through borrowing, debt, or asset sales.

Figure 5 demonstrates an apparent discrepancy between median reported monthly household income (₦100,000) and median routine household expenditures (₦155,000). Because these values represent independently calculated cohort-level medians, their difference should not be interpreted as a uniform ₦55,000 monthly deficit across individual households. Nevertheless, the finding is consistent with the substantial baseline economic vulnerability observed in this cohort, with 97.0% of households with sufficient baseline data already below the specified poverty threshold before OOP healthcare payments. The apparent discrepancy may also reflect variability or underreporting of income, informal or non-cash sources of support, borrowing, or other household financing mechanisms. Because reported household income is used as the denominator for the CHE10% calculation, potential underreporting of income could result in overestimation of CHE using this threshold.

Taken together, these findings highlight a significant disconnect between the cost of neurosurgical treatment and the financial capacity of patients and their families, even within a public hospital setting. The composition of costs in our cohort highlights critical gaps. The high cost of care was primarily driven by direct medical expenses, with a median of ₦969,000 ($633; IQR: ₦507,342–₦1,457,635), including surgical procedures, diagnostics, medications, and consumables–costs that reflect broader systemic gaps in neurosurgical infrastructure, equipment, and critical care capacity previously documented in Nigeria and Uganda (9, 21, 22). However, direct non-medical expenses were also substantial, with a median of ₦202,000 ($132; IQR: ₦97,750–₦308,700), and covered items such as transportation, meals, and in-hospital necessities. These findings align with previous research which noted that non-medical costs often impose a significant financial burden, particularly for patients referred to tertiary centers from distant locations (23).

Additionally, patients and caregivers missed a median of 19 and 20 days of work or domestic responsibilities, respectively, often without compensation. This aligns with findings in other neurosurgical settings, where caregiving after procedures such as craniotomy or microdiscectomy has been shown to impose substantial financial burdens, including loss of income and additional OOP costs (24). More than half of the households borrowed money to pay for care, and about 10% sold property to raise funds. Similar patterns have been observed in other LMICs, where borrowing, asset sales, and job losses are frequent coping strategies in response to high surgical costs. In a Ugandan study, 53% of households borrowed money, 21% sold possessions, and 17% lost employment due to hospitalization (25). These coping mechanisms not only reflect the absence of effective financial risk protection but also further exacerbate household vulnerability and debt.

Delays in care further compounded costs. Previous studies in Nigeria have identified long transportation distances and lack of funds as leading causes of delayed neurosurgical presentation (7, 26). Our finding that over a quarter of patients traveled >50 km (often on multiple transport legs) suggests high indirect cost burdens even before surgery. Late presentation not only worsens outcomes but may extend hospital stays and expenses, creating a vicious cycle of poverty and illness.

From a policy perspective, our results highlight urgent needs to strengthen financial protection mechanisms for patients requiring essential neurosurgical services. Nigeria’s current healthcare financing structure is severely constrained. In 2022, 76.1% of all healthcare expenditure was paid out-of-pocket by households in Nigeria, while domestic general government health expenditure accounted for only 0.62% of GDP and just 14.5% of total health expenditure (1). Government health spending per capita was only $13 USD, far below the recommended minimum of $86 USD per capita suggested by the World Health Organization to deliver essential health services in low-income countries (1, 27). In comparison, the average government health expenditure per capita in sub-Saharan Africa was approximately $34 USD in the same period (1). This heavy reliance on OOP payments limits access to care and increases the risk of catastrophic and impoverishing health expenditures.

Lessons from health system reform in other countries underscore the importance of risk pooling and prepayment: for example, expanding social insurance or tax-based funding can dramatically reduce OOP burden. As emphasized by Mathauer et al. and Kodali, advancing universal health coverage (UHC) requires strengthening prepaid, pooled financing mechanisms and ensuring government commitment to protect vulnerable populations (28, 29). In addition, Koohpayehzadeh et al. suggest that targeted subsidies for high-cost surgical services, such as neurosurgery, and inclusion of direct non-medical and indirect expenses are essential to alleviate financial strain on households (30). For Nigeria, this could involve scaling up NHIS enrollment beyond the formal sector and explicitly including expensive surgical services. Targeted financial aid, such as subsidizing neurosurgical interventions or covering patient transportation, would directly alleviate the burdens we observed. Decentralizing neurosurgical services and improving referral pathways and coordination could reduce travel costs and care delays.

### Strengths and limitations

This is the first comprehensive study of neurosurgical patient costs and attendant socioeconomic consequences in Nigeria. We used a prospective, multi-timepoint design to capture real-world expenses and socioeconomic impacts across the care continuum. By including indirect costs and caregiver perspectives, we present a holistic view of the burden. However, there are limitations. The observational design cannot prove causality or capture unmeasured factors. Further, the single-center setting may limit generalizability, given the size, population, and geopolitical diversity of the country. Moreover, UCH Ibadan is a high-volume national center, so costs might differ in smaller hospitals or private clinics. We, also, likely under-sampled the most critically ill, who could not be interviewed despite our inclusion of caregivers, possibly underestimating costs for that subgroup. Recall bias is possible, though mitigated by multiple survey points. Self-reported household income and expenditure may also be subject to reporting error, particularly in households with variable or informal sources of income. Future studies should consider following households longitudinally to assess long-term financial recovery and the full economic impact over time. Despite these, our findings provide valuable baseline data for policy discussions.

## Conclusion

Neurosurgical care in Nigeria’s public hospitals imposes extreme economic hardship on patients and families. Almost every household in our study faced catastrophic health spending, with OOP expenditure substantially deepening pre-existing poverty among already economically vulnerable households. High direct medical fees, combined with substantial non-medical costs, drive these outcomes. Expanding financial protection is imperative: greater government health spending and expanded insurance (or national financing schemes) are needed to cover essential surgical services. Subsidies or support for direct and indirect neurosurgical expenses could prevent families from financial ruin. As Nigeria strives toward UHC, ensuring equitable access to life-saving neurosurgical interventions must be a priority. Our results can inform policymakers and health planners about the scope of the problem, and underscore that without reform, vulnerable households will continue to bear the hidden cost of neurosurgical care.

## Data Availability

The original contributions presented in the study are included in the article/supplementary material, further inquiries can be directed to the corresponding author.

## Appendix

Appendix 1 Full details of medical expenditure reported by participants.

**Table:**

Expense	*n* (%)	Median (₦) (Q1, Q3)
Direct medical costs		
Consumables	99 (100.0)	60,000 (30,000, 200,000)
Drugs	97 (98.0)	150,000 (64,000, 250,000)
Laboratory tests	96 (97.0)	53,500 (39,777, 150,000)
Procedure fees	81 (81.8)	15,000 (10,000, 20,000)
Surgery fee	53 (53.5)	280,000 (249,000, 441,000)
Anesthesia fee	50 (50.5)	140,000 (90,000, 141,750)
Rehabilitation fee	48 (48.5)	36,000 (18,000, 80,000)
Devices	29 (29.3)	110,000 (25,000, 140,000)
ICU fee	16 (16.2)	180,000 (122,290, 338,750)
Post-discharge medications	66 (66.7)	50,000 (25,000, 100,000)
Overall direct medical cost	99 (100)	969,000 (507,342, 1,457,635)
Direct non-medical costs		
Toiletries	94 (94.9)	30,000 (15,000, 50,000)
Food (while hospitalized)	94 (94.9)	69,500 (40,000, 150,000)
Transportation cost	88 (88.9)	50,000 (20,000, 90,000)
Food while in transit	81 (81.8)	15,000 (10,000, 42,000)
Administrative fee	2 (2.0)	78,000 (42,000, 114,000)
Overall direct non-medical cost	95 (96)	202,000 (97,750, 308,700)
Indirect costs		
Income lost by caregiver	81 (81.8)	108,000 (24,000, 300,000)
Income lost by patient	64 (64.6)	100,524 (46,368, 191,803)
Overall indirect cost	88 (88.9)	141,639 (41,902–539,180)

₦, Nigerian Naira; Q1, Lower quartile; Q2, Upper quartile.
